# Supplementary material for: Causal Relationship between Aging and Anorexia Nervosa: A White-Matter-Microstructure-Mediated Mendelian Randomization Analysis
Source: Biomedicines. 2024 Aug 16;12(8):1874. doi: 10.3390/biomedicines12081874 (PMC11351342; doi:10.3390/biomedicines12081874)
Supplement: Supplementary file 1 [file biomedicines-12-01874-s001.zip › Supplementary Figure/Figure S4/Singe SNP plot of MR analysis for TL to ubm-b-2097.pdf]

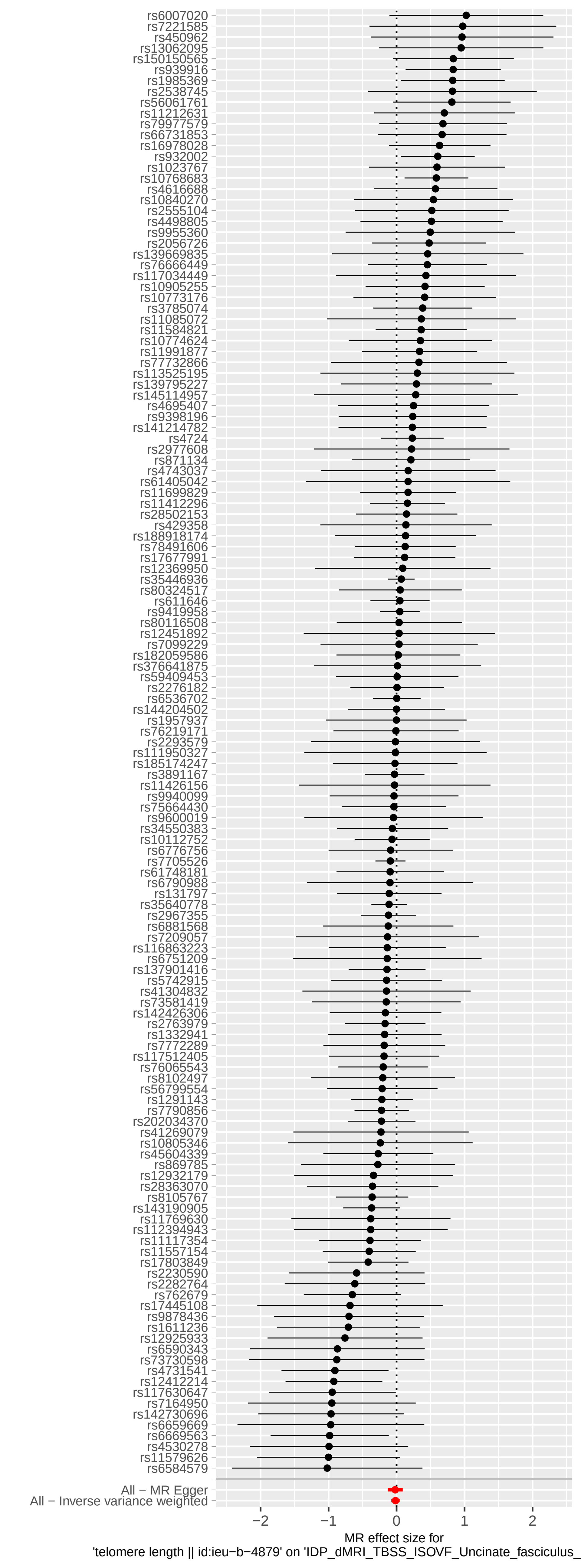
rs6007020
rs7221585
rs450962
rs13062095
rs150150565
rs939916
rs1985369
rs2538745
rs56061761
rs11212631
rs79977579
rs66731853
rs16978028
rs932002
rs1023767
rs10768683
rs4616688
rs10840270
rs2555104
rs4498805
rs9955360
rs2056726
rs139669835
rs76666449
rs117034449
rs10905255
rs10773176
rs3785074
rs11085072
rs11584821
rs10774624
rs11991877
rs77732866
rs113525195
rs139795227
rs145114957
rs4695407
rs9398196
rs141214782
rs4724
rs2977608
rs871134
rs4743037
rs61405042
rs11699829
rs11412296
rs28502153
rs429358
rs188918174
rs78491606
rs17677991
rs12369950
rs35446936
rs80324517
rs611646
rs9419958
rs80116508
rs12451892
rs7099229
rs182059586
rs376641875
rs59409453
rs2276182
rs6536702
rs144204502
rs1957937
rs76219171
rs2293579
rs111950327
rs185174247
rs3891167
rs11426156
rs9940099
rs75664430
rs9600019
rs34550383
rs10112752
rs6776756
rs7705526
rs61748181
rs6790988
rs131797
rs35640778
rs2967355
rs6881568
rs7209057
rs116863223
rs6751209
rs137901416
rs5742915
rs41304832
rs73581419
rs142426306
rs2763979
rs1332941
rs7772289
rs117512405
rs76065543
rs8102497
rs56799554
rs1291143
rs7790856
rs202034370
rs41269079
rs10805346
rs45604339
rs869785
rs12932179
rs28363070
rs8105767
rs143190905
rs11769630
rs112394943
rs11117354
rs11557154
rs17803849
rs2230590
rs2282764
rs762679
rs17445108
rs9878436
rs1611236
rs12925933
rs6590343
rs73730598
rs4731541
rs12412214
rs117630647
rs7164950
rs142730696
rs6659669
rs6669563
rs4530278
rs11579626
rs6584579
All − MR Egger
All − Inverse variance weighted
−2
−1
0
1
2
MR effect size for
'telomere length || id:ieu−b−4879' on 'IDP_dMRI_TBSS_ISOVF_Uncinate_fasciculus_
